# Clinical Presentation and Management of Multisystem Inflammatory Syndrome in Children With COVID-19: A Systematic Review

**DOI:** 10.7759/cureus.46918

**Published:** 2023-10-12

**Authors:** Mohammed H Albanji, Ahmed A Baghafar, Yasser A Alghanmi, Mohammed M Shaaban, Ebrahim A Alkashlan, Haifa H Sende, Mofareh S Alzahrani, Nuha N Filfilan

**Affiliations:** 1 Pediatric Neurology, Royal Commission Medical Center, Yanbu, SAU; 2 Pediatrics, Royal Commission Medical Center, Yanbu, SAU; 3 Family and Community Medicine, College of Medicine, Taif University, Taif, SAU

**Keywords:** toxic shock-like syndrome, kawasaki-like multisystem inflammatory disease, mis-c, post-covid-19 hyperinflammatory syndrome, covid-19, multisystem inflammatory syndrome in children

## Abstract

Multisystem inflammatory syndrome in children (MIS-C) is a relatively new syndrome associated with coronavirus disease 2019 (COVID-19) that is characterized by a severe clinical course compared to pediatric COVID-19. This review aimed to compile the available evidence on the clinical presentation and management of MIS-C in children with COVID-19. During this systematic review, a comprehensive search was performed in the following databases: PubMed, Embase, Medline, Google Scholar, Cochrane, and Scopus, using predetermined search terms, such as Medical Subject Headings (MeSH) and keywords to find relevant studies on the MIS-C. Relevant data were extracted, and the quality of the studies was evaluated using suitable methods. The collected findings were synthesized and discussed in the study.

The World Health Organization's (WHO) definition of MIS-C was the most favored due to its precision and inclusiveness. MIS-C primarily affected children aged 6-12 years, with male predominance. MIS-C involves a range of systems, including gastrointestinal, cardiovascular, hematologic, mucocutaneous, and respiratory. Radiographic findings revealed cardiovascular abnormalities, solid visceral organ involvement, and bowel abnormalities, reflecting a systemic inflammatory process. Laboratory investigations unveiled elevated inflammatory markers, neutrophil activation, release of extracellular traps in vessels, elevated procalcitonin, hyponatremia, hypoalbuminemia, low hemoglobin, and thrombocytopenia. The inflammatory markers and autoantibody profiles are essential in differentiating MIS-C from COVID-19. The preferred treatment primarily involves immunomodulatory therapies like intravenous immunoglobulin (IVIG), glucocorticoids, and interleukin-6 or 1RA inhibitors or a combination of those. In severe cases, extracorporeal membrane oxygenation (ECMO) and mechanical ventilation are necessary, leading to reduced mortality and quick recovery. This review found that the average hospital stay was seven days, and most discharged children fully recovered within seven days.

MIS-C is a life-threatening post-COVID-19 condition and involves multiple systems due to systemic inflammation, with elevated inflammation markers. Recognition of multisystem involvement is crucial, and prompt identification and multidisciplinary treatment are vital for optimal outcomes.

## Introduction and background

The emergence of the novel coronavirus disease 2019 (COVID-19) caused by the severe acute respiratory syndrome coronavirus 2 (SARS-CoV-2) has led to a global health crisis with significant impacts on populations worldwide [1,2]. While initial reports primarily highlighted that most children with COVID-19 are asymptomatic or mildly to moderately affected, almost 2-4% of children could be critically ill [3,4]. In May 2020, a novel syndrome characterized by a severe systemic inflammatory response that impacts multiple organs in COVID-19-infected children was termed multisystem inflammatory syndrome in children (MIS-C) [5]. The first cases of this syndrome were initially reported in the United Kingdom (UK) [2], Italy [6], and the United States [7]. This novel syndrome has raised significant concern among healthcare professionals and parents alike as it shares its characteristic manifestations with Kawasaki syndrome, Toxic-shock syndrome, secondary hemophagocytic lymphohistiocytosis (SHLH), and macrophage activation syndrome (MAS) [6]. MIS-C is a rare but severe condition that primarily affects children and adolescents infected with or exposed to SARS-CoV-2, the virus causing COVID-19 [4,8]. 

The clinical presentation of MIS-C involves a persistent fever lasting more than 24 hours and other symptoms such as gastrointestinal disturbances, skin rash, and conjunctivitis [8]. Although respiratory symptoms may or may not be present, the syndrome can progress rapidly, leading to acute cardiac dysfunction, shock, and multiorgan failure. MIS-C is diagnosed based on clinical criteria, including fever, inflammation markers (C-reactive protein (CRP), erythrocyte sedimentation rate (ESR), interleukin-6 (IL-6)), and evidence of a recent SARS-CoV-2 infection [9,10]. Management involves a multidisciplinary approach with hospitalization in the pediatric intensive care unit (PICU) for close monitoring [7,8,11]. Treatment should aim to control inflammation using intravenous immunoglobulin (IVIG), corticosteroids, and immunomodulatory agents like tocilizumab. Cardiac support, including inotropic agents and extracorporeal membrane oxygenation (ECMO), may be necessary [12], and prophylactic anticoagulation is considered due to the increased risk of thrombosis [8,12]. 

Although relatively rare, MIS-C represents a unique and challenging manifestation of COVID-19 and can lead to severe complications and even mortality if not promptly recognized and managed. Understanding the clinical presentation and adopting a multidisciplinary approach to its management is essential for ensuring optimal outcomes in affected children. As research evidence continues to emerge about this syndrome, ongoing research is essential to improve understanding and develop better strategies for the prevention and treatment of MIS-C. Therefore, this systematic review aimed to compile available evidence on the clinical presentation and management of MIS-C in children with COVID-19.

## Review

Methods

The following research question guided this systematic review: "What is the clinical presentation of MIS-C with COVID-19, and what are the effective management strategies for this condition?"

Search Strategy

The review was performed according to the preferred standards of systematic reviews and meta-analysis reporting (PRISMA) [13]. We comprehensively and systematically searched the following databases: PubMed, Embase, Medline, Google Scholar, Cochrane, and Scopus. The search terms were chosen, combining medical subject headings (MeSH terms) and keywords using Boolean operators ("AND," "OR") to create relevant search queries. The terms and keywords related to MISC-C and COVID-19 were used in searching: "Novel coronavirus" OR "2019-novel coronavirus" OR "Novel CoV" OR "2019-nCoV" OR "2019-CoV" OR "COVID-19" OR "COVID 19" OR "SARS-CoV-2" OR "Middle East Respiratory Syndrome" OR "MERS" OR "MERS-CoV" OR "Severe Acute Respiratory Syndrome" OR "SARS" OR "SARS-CoV" OR "SARS Related" OR "SARS-Associated" AND " multisystemic inflammatory syndrome" OR" MIS-C" OR "MIS" OR "Multisystem Inflammatory Syndrome in Children" OR "Kawasaki-like multisystem inflammatory disease" OR "Inflammatory Multisystem Syndrome" OR "Multisystem Inflammatory Syndrome" OR "Toxic shock-like syndrome" OR "hyperinflammatory response" OR "Atypical Kawasaki." We searched the reference lists of the identified articles to find any additional studies that might be relevant. Only studies conducted and published in English within the past three years were considered to incorporate the latest research evidence on MIS-C.

Inclusion and Exclusion Criteria

We included studies that satisfied the following criteria: (1) Primary research studies (observational, cohort, case-control, and clinical trials), systematic reviews, and meta-analyses that focus on the clinical presentation and/or management of MIS-C in children with COVID-19; (2) Studies with a clear definition of MIS-C, including diagnostic criteria and clinical features; (3) Studies reporting on management strategies, including pharmacological and non-pharmacological interventions; and (4) Studies with a pediatric population (age ≤ 18 years). Studies were excluded if they were reviews, theses, editorials, letters to the editor, commentary, opinion articles, narrative and scoping reviews, case reports, and any other article published in non-peer-review journals.

Study Selection 

**Figure 1:**
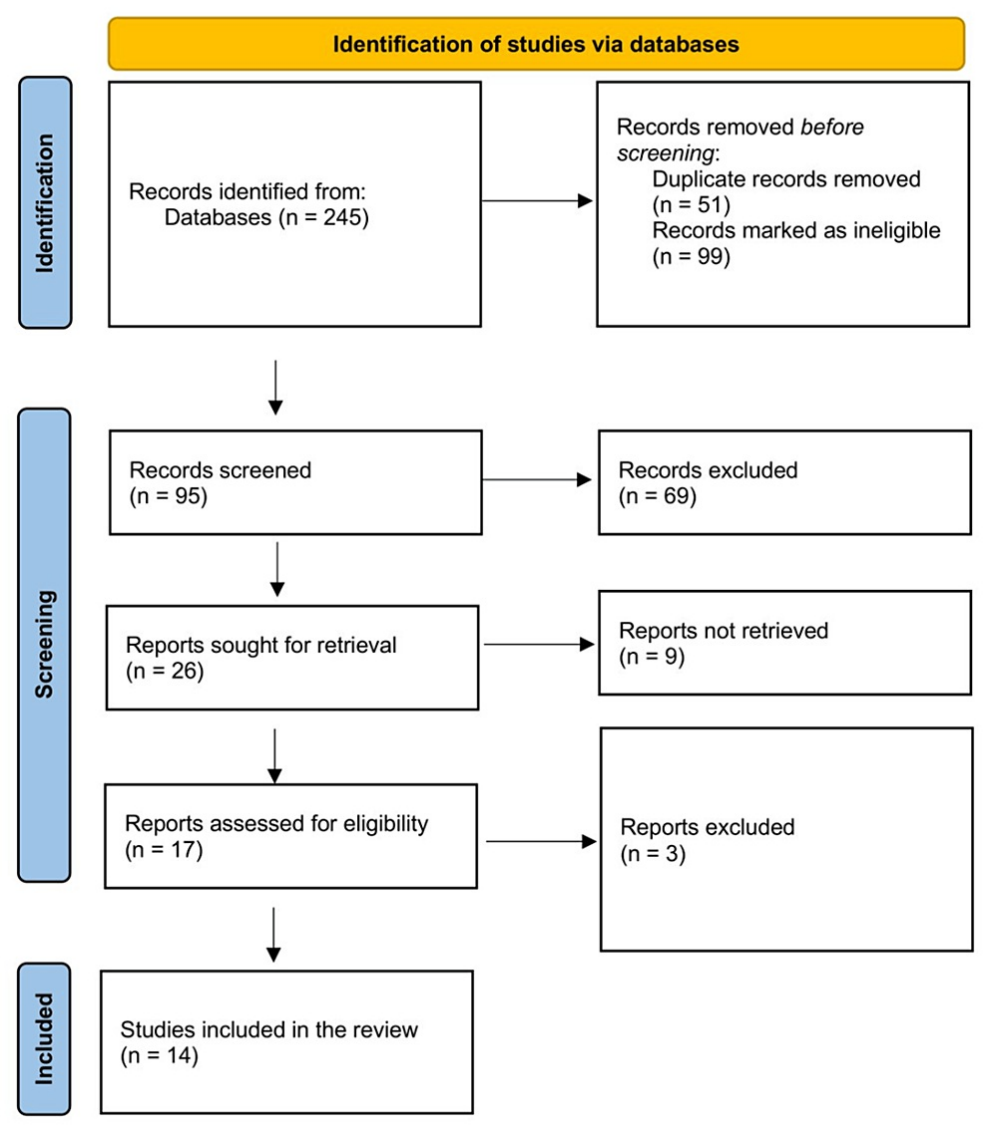
PRISMA flow diagram showing the selection process. PRISMA: preferred standards of systematic reviews and meta-analysis.

Two reviewers (MHA and AB) screened the abstracts and titles independently and included the eligible articles. Then, both authors reviewed the full text for eligibility, and any discrepancy was solved by discussion, with a third author (YG) involved when necessary. The reviewers first screened all titles and abstracts with the pre-defined criteria and categorized the articles into three groups: eligible, not eligible, and unclear. In the second step, the full texts of the potentially eligible or unclear studies were thoroughly reviewed for a final decision on inclusion. The selection process is further detailed in Figure 1.

Data Extraction and Quality Assessment

A structured data extraction form was used to extract data from the reports of all included studies independently by all authors. Discrepancies in the extracted data were resolved through discussion. When available, the following data were extracted from each selected study: First author, publication year, study design, and summary of findings.

We evaluated the quality of the included studies using appropriate tools specific to their study design. The Newcastle-Ottawa Scale (NOS) [14] was used to assess the quality of cohort studies, with a scale of 0 to 9. Each NOS item, except comparability, could only receive one point, resulting in a minimum score of zero [14]. For intervention studies, we utilized the Cochrane Collaboration's Risk of Bias tool (CCRB) [15], which consists of seven components, including random sequence generation, allocation concealment, blinding of participants and personnel, blinding of outcome assessment, completeness of outcome data, selective outcome reporting, and other biases. The risk of bias for each item was graded as low, high, or uncertain [15].

To assess the quality of cross-sectional research, we used the Agency for Healthcare Research and Quality Scale (AHRQ) [16], which assigns scores ranging from 0 to 11. Higher ratings indicate better research methodology. All reviewers independently evaluated the risk of bias in the studies, and in cases of discrepancies, a third reviewer was consulted for assistance.

Data Synthesis and Analysis 

Due to heterogeneity across the included papers, a meta-analysis was not feasible. Instead, we synthesized the findings by summarizing and categorizing them based on major themes related to the clinical presentation and management of MIS-C. Considering the strength of evidence, consistency of findings, and limitations of the included studies, we discussed the clinical presentation and management of MIS-C reported in the included studies.

Ethical Considerations 

This was a systematic review of previously published studies that did not directly involve participants. As a result, no ethical approval was required.

Results

We initially got 245 titles and abstracts from the database search. After removing duplicates, and initial screening for eligibility, 105 articles' titles and abstracts were legible for the full-text evaluation. A thorough screening of full-text articles resulted in 14 articles meeting the inclusion criteria (Table 1). Of the included studies, seven studies were retrospective observational studies, while one was a prospective observational study. One study was both prospective and retrospective surveillance; one used systems-level analyses; one was a case-control study; one was an ambispective multicenter cohort, another one was a retrospective cohort, and the last one was a systematic review.

**Table 1:** Characteristics of the included studies. MIS-C: multisystem inflammatory syndrome in children; COVID-19: coronavirus disease; SARS-CoV-2: severe acute respiratory syndrome coronavirus 2; RA: receptor antagonist; LMWH: low molecular weight heparin; CI: confidence interval; KDIGO: kidney disease improving global outcomes.

Authors	Year of publication	Title	Study design	Findings
Consiglio et al. [17]	2020	The immunology of multisystem inflammatory syndrome in children with COVID-19	Systems-level analyses	The findings indicated that the inflammatory response observed in MIS-C was distinct from the cytokine storm seen in severe acute COVID-19. Instead, it was similar to Kawasaki disease. However, there were differences, particularly concerning T cell subsets, interleukin (IL)-17A, and biomarkers associated with arterial damage. Furthermore, based on autoantibody profiling, it appeared that multiple autoantibodies might play a role in the development of MIS-C.
Boribong et al. [18]	2022	Neutrophil profiles of pediatric COVID-19 and multisystem inflammatory syndrome in children	Case-control	Findings revealed that the hyperinflammatory presentation in MIS-C may be mechanistically linked to persistent SARS-CoV-2 antigenemia, driven by uncontrolled neutrophil activation and neutrophil extracellular traps release in the vasculature.
Feldstein et al. [19]	2020	Multisystem inflammatory syndrome in U.S. children and adolescents	Prospective and retrospective surveillance	This study included 186 MIS-C patients, with a median age of 8.3 years, and 62% were male. Most were previously healthy, and 70% tested positive for SARS-CoV-2. Organ-system involvement was widespread, including gastrointestinal (92%), cardiovascular (80%), hematologic (76%), mucocutaneous (74%), and respiratory (70%) systems. Hospitalization lasted a median of seven days, with 80% receiving intensive care, 20% on mechanical ventilation, and 2% fatalities. Coronary-artery aneurysms were observed in 8%, and Kawasaki's disease-like features in 40%. Nearly all patients had elevated inflammation biomarkers. Immunomodulating therapies were common, with 77% receiving intravenous immune globulin, 49% receiving glucocorticoids, and 20% receiving interleukin-6 or 1RA inhibitors.
Miller et al. [7]	2022	Multisystem inflammatory syndrome in children—United States, February 2020–July 2021	Retrospective observational study	Out of the 4470 cases included, the median patient age increased over time, with a median age of nine years during the third wave. Male predominance also increased in the third wave. There was a significant increase in severe hematologic and gastrointestinal involvement across the study period. However, the frequency of several cardiovascular complications and renal failure declined. The provision of critical care, including mechanical ventilation and extracorporeal membrane oxygenation (ECMO), decreased, as did the duration of hospitalization and mortality.
Hoste et al. [4]	2021	Multisystem inflammatory syndrome in children related to COVID-19: a systematic review	Systematic review	MIS-C predominantly affected males (58.9%) and ethnic minorities (37.0%, Black) at a median age of eight years. Comorbidities were rare, except for obesity (25.3%). MIS-C was characterized by fever (99.4%), gastrointestinal (85.6%), and cardiocirculatory manifestations (79.3%), along with elevated inflammatory biomarkers. Surprisingly, 50.3% also present respiratory symptoms. The shock was observed in 56.3% of cases, necessitating intensive care treatment, including ECMO, in 3.8%. Despite its severity, mortality remains low (1.9%). The WHO definition was preferred in most cases, given its precision and inclusiveness.
Levy et al. [20]	2022	Multisystem inflammatory syndrome in children by COVID-19 vaccination status of adolescents in France	Retrospective observational study	Between September 1 and October 31, 2021, 107 children with MIS-C were hospitalized, and 31% were adolescents eligible for vaccination. These adolescents had a median age of 13.7 years, with 81% male and 88% admitted to a PICU. None of the adolescents had been fully vaccinated, but seven had received one dose. The hazard ratio for MIS-C after the first vaccine dose compared to unvaccinated adolescents was 0.09 (95% CI, 0.04-0.21; P).
Brooks et al. [21]	2023	Multisystem inflammatory syndrome in children associated with COVID-19 presents as cervical inflammation	Retrospective observational study	Findings showed that an ill-appearing child displaying symptoms or signs of cervical inflammation should be evaluated for MIS-C. Among 37 children diagnosed with MIS-C, 13.5% presented with cervical symptoms, and later, they all developed the full MIS-C phenotype.
Diorio et al. [22]	2020	Multisystem inflammatory syndrome in children and COVID-19 are distinct presentations of SARS-CoV-2	Prospective observational study	Children infected with SARS-CoV-2 are at risk of developing MIS-C. Cytokine profiling and peripheral blood smear examination may help identify between patients with MIS-C and those with severe COVID-19.
Kapoor et al. [9]	2022	Multisystem inflammatory syndrome in children (MIS-C) related to SARS-CoV-2 and one-year follow-up	Retrospective observational study	The majority of children with MIS-C had gastrointestinal involvement (92%), followed by cardiovascular (85%), central nervous system (CNS) (74%), respiratory (72%), mucocutaneous (59%), and renal (31%) involvement. Hypotension was the initial symptom in 43% of cases. Procalcitonin levels were elevated in all patients, BNP in 31.5%, and 83.3% had a low ejection fraction. Radiographs were abnormal in 59%. All children tested positive for anti-SARS-CoV-2 antibodies and negative for cultures. Treatment included methylprednisolone or IVIg in 77%, mechanical ventilation in 18.5%, inotropic support in 77%, aspirin in 48%, and LMWH in 54%. At seven-day follow-up, 98% had normal echocardiography; at six months and one year, all children had normal echocardiography.
Magboul et al. [23]	2023	Multisystem inflammatory syndrome in children (MIS-C) related to COVID-19 infection in the state of Qatar: Association with Kawasaki-like Illness	Retrospective observational study	Most patients exhibited involvement of four or more organ systems. Nine out of the 15 presented children had Kawasaki disease-like symptoms, and all had gastrointestinal symptoms. Laboratory investigations showed elevated D-dimer, hyponatremia, and hypoalbuminemia in all patients. Low hemoglobin, thrombocytopenia, and sterile pyuria were observed in 86.6%, 60%, and 75% of cases, respectively. Treatment included a combination of intravenous immunoglobulin and corticosteroids, and in some refractory cases, immunomodulatory agents like Anakinra were used.
Blumfield et al. [24]	2021	Imaging findings in multisystem inflammatory syndrome in children (MIS-C) associated with coronavirus disease (COVID-19)	Retrospective observational study	Radiographic findings of MIS-C primarily showed cardiovascular abnormalities, but they also showed solid visceral organ, gallbladder, and bowel abnormalities, along with ascites, indicating a widespread inflammatory process affecting multiple systems in the body. Most abnormalities were cardiomegaly (75%), pleural effusion (63%), and atelectasis (63%).
Antúnez-Montes et al. [25]	2020	COVID-19 and multisystem inflammatory syndrome in Latin American children: a multinational study	Ambispective multicentric cohort	Among 409 children studied, 23.2% were diagnosed with MIS-C, and 12.7% required admission to a pediatric intensive care unit (PICU). 22.5% of patients required oxygen therapy, 2% on continuous positive airway pressure (CPAP), 7% on mechanical ventilation, and 8.5% on inotropic support. Factors associated with PICU admission were pre-existing medical conditions, immunodeficiency, lower respiratory tract infection, gastrointestinal symptoms, radiological changes suggestive of pneumonia and acute respiratory distress syndrome, and low socioeconomic conditions.
Feldstein et al. [19]	2020	Characteristics and outcomes of US children and adolescents with multisystem inflammatory syndrome in children (MIS-C) compared with severe acute COVID-19	Prospective observational study	Comparatively, MIS-C patients were more prevalent in the age group of six to 12 years (40.8% vs 19.4%), were non-Hispanic Black (32.3% vs 21.5%), and had higher instances of severe cardiovascular (56.0% vs 8.8%) or mucocutaneous involvement (7.1% vs 2.3%), as well as elevated inflammation levels when compared to those with severe COVID-19.
Lipton et al. [26]	2021	AKI in COVID-19–associated multisystem inflammatory syndrome in children (MIS-C)	Retrospective cohort	Among the 57 children with MIS-C, 46% were diagnosed with acute kidney injury (AKI). Most (58%) were classified as having KDIGO stage 1 AKI. All patients with AKI recovered, and 61% recovered by day two. Only one patient required dialysis. The AKI cohort was older (P<0.001) and had higher median peak values of CRP (P<0.001), IL-6 (P<0.001), ferritin (P<0.001), procalcitonin (P=0.002), left ventricular systolic dysfunction (P<0.001), and lymphopenia (P=0.01) when compared to those without AKI.

As reported by a systematic review, in most cases, the World Health Organization (WHO) definition of MIS-C was preferred, given its precision and inclusiveness [4]. The same definition was used in other studies included in our review. Demographically, MIS-C was reported predominantly among males and children aged 6-12-year-old children in four studies [7,19,20,27]. One study reported that 81% of adolescents were male, and the median age was 13.7 years, aligning with other studies showing male predominance. However, another study showed that MIS-C patients had a median age of 8.3 years [19], and another one reported increased MIS-C cases in the third wave of COVID-19 [7].

This systematic review showed that children infected with SARS-CoV-2 are at risk of MIS-C [21,22,25]. One study on 54 children with MIS-C showed that all of them tested positive for anti-SARS-CoV-2 antibodies [9]. Some included studies showed that MIS-C patients tend to be admitted to the PICU and need CPAP and oxygen supply, indicating a severe life-threatening illness [20,25]. Cervical inflammation was suggested to characterize MIS-C. One study showed that 13.5% of MIS-C cases had cervical inflammation [21]. Factors associated with PICU admission were pre-existing comorbidities, immunodeficiency, lower respiratory tract infection, gastrointestinal symptoms, radiographic changes suggestive of pneumonia and acute respiratory distress syndrome, and low socioeconomic conditions [25]. Six studies reported that gastrointestinal, cardiovascular, including coronary-artery aneurysms, hematologic, mucocutaneous, and respiratory systems were involved in over 70-92% of MIS-C patients [4,7,9,23,24,27]. 

One article reported the central nervous system in 74% of patients [9], while another study evaluating radiographic findings reported cardiomegaly (75%), pleural effusion (63%), and atelectasis (63%) as the most common findings in MIS-C patients [24]. Another one showed that radiographic findings showed cardiovascular, solid visceral organ, gallbladder, and bowel abnormalities, along with ascites, indicating a widespread inflammatory process [24]. Most MIS-C patients have comorbidities involving four or more organs and Kawasaki disease-like symptoms [23]. On the other hand, one study showed that MIS-C patients have higher rates of severe cardiovascular (56.0% vs 8.8%) or mucocutaneous involvement (7.1% vs 2.3%), as well as elevated inflammation levels when compared to those with severe COVID-19 [19], aligning with another study showing elevated inflammatory biomarkers and fever as characteristics of MIS-C [4,19]. 

Cytokine profiling and peripheral blood smear examination were suggested to help identify between patients with MIS-C and those with severe COVID-19 [22]. Studies that evaluated biomarkers in relation to MIS-C indicated that MIS-C's inflammatory response differs from the cytokine storm in severe COVID-19, while sharing similarities with Kawasaki disease, albeit with discrepancies in T cell subsets, IL-17A, and artery damage biomarkers [17]. Autoantibody profiling suggested multiple autoantibodies might contribute to MIS-C development [18]. Studies also showed that hyperinflammatory MIS-C presentation might stem from sustained SARS-CoV-2 antigenemia, involving unchecked neutrophil activation and the release of extracellular traps in vessels [18]. Procalcitonin was high in all MIS-C patients [9]. One study found that brain natriuretic peptide (BNP) in 31.5% and 83.3% of all MIS-C patients showed reduced ejection fraction, indicating cardiovascular involvement, particularly heart failure [9]. Further lab analyses revealed D-dimer elevation, hyponatremia, and hypoalbuminemia in all MIS-C patients. In addition, 86.6% had low hemoglobin, 60% thrombocytopenia, and 75% sterile pyuria.

Though rarely reported, renal involvement might happen [7]. One study showed renal involvement in 31% of MIS-C cases, which quick recovery [9]. However, another study showed that among the 57 children with MIS-C study, 46% were diagnosed with acute kidney injury (AKI), with 61% recovering by day 2 of diagnosis. The MIS-C patients who were older (P<0.001) had higher median peak values of CRP (P<0.001), IL-6 (P<0.001), ferritin (P<0.001), procalcitonin (P=0.002), left ventricular systolic dysfunction (P<0.001) and lymphopenia (P=0.01) when compared to those without AKI [26].

The most common mode of treatment reported was immunomodulating therapies, such as IVIG, glucocorticoids, and interleukin-6 or 1RA inhibitors [4,7,9,19,23]. The combination of IVIG and glucocorticoids, such as methylprednisolone, was reported [23]. ECMO, mechanical ventilation, and inotropic support were mostly used for critically ill children [7]. One study reported the use of low molecular weight heparin (LMWH) in 54% of patients [9]. Though one study reported shock among 56.3% of cases of MIS-C, the mortality rate remained low (1.9%) [4]. As reported in another study, mechanical ventilation, ECMO, and ionotropic therapy contributed to decreased hospitalization and mortality [7]. On average, hospitalization lasted seven days, and 98% of all discharged children fully recovered in the other seven days [9,19].

Discussion

MIS-C is a rare but potentially life-threatening condition that predominantly affects children and adolescents infected with SARS-CoV-2 or recent exposure to the virus [10,11]. Though it presents after the acute phase of COVID-19, understanding of this syndrome remains poor and is still relatively new for healthcare providers [28]. Therefore, this systematic review would enhance the knowledge of the clinical presentation and effective management of MIS-C to help healthcare providers expedite diagnosis, initiate prompt treatment, and improve patient outcomes.

The findings showed that the WHO definition of MIS-C post-COVID-19 infection was the most used for diagnosing children with this syndrome. This definition encompasses the following criteria: Febrile children and adolescents aged 0-19 with three-day fever, with two (1) rash or bilateral non-purulent conjunctivitis or mucocutaneous inflammation signs (oral, hands, or feet), (2) hypotension or shock, (3) myocardial dysfunction, pericarditis, valvulitis, or coronary abnormalities, (4) features of coagulopathy, (5) gastrointestinal problems, with elevated inflammatory markers and positive COVID-19 test or likely positive contact [29]. These criteria have similarities to the suggested criteria of the US Center for Disease Control and Prevention (CDC) [30,31].

Previous studies showed that MIS-C mostly affects children aged 1-20 years, with a mean range of 9-12 years [32], similar to the age range reported in our systematic review. Though our findings did not evaluate the age range of patients with MIS-C compared to patients with COVID-19, a previous systematic review showed no difference in the age means noticed in patients with MIS-C compared to COVID-19 pediatric patients [33]. Aligning with our findings, previous studies found that male children were the most affected by MIS-C along with black children [33,34], though the ethnicity difference was only reported in studies from the United States of America (USA). Despite the mild clinical course of COVID-19 in children, the MIS-C clinical course is more severe. Previous studies reported that almost half of COVID-19 patients need ICU admission, of which 20% require mechanical ventilation [35]. In contrast, our findings showed that most MIS-C patients require PICU admission and mechanical ventilation (up to 88% and 20%, respectively). This might be attributed to the widespread inflammatory process involving multiple organs, including the CNS, gastrointestinal organs, heart, and respiratory organs. Aligning with our findings, research has shown that gastrointestinal symptoms and cardiovascular symptoms are among the most common in MIS-C patients [36]. Though our included studies did not report skin rash among typical signs of MIS-C, there is evidence showing that skin manifestation is typical for MIS-C, the same for Kawaki disease and COVID-19 [36]. However, we found neurological symptoms among the signs of MIS-C, aligning with previous research showing that MIS-C neurological involvement is more common and severe in MIS-C than in COVID-19 [37]. While the mortality rate of COVID-19 among pediatric patients was reported to range between 0.21% and 3.3% [38-40], our findings showed the mortality rate of MIS-C to be 1.9%, which is in the range of the COVID-19 mortality rate in children. This is consistent with another study showing that the mortality rate of MIS-C is similar to that of COVID-19 in this patient population [33], despite the latter being more severe. 

Compared to other diseases with similar clinical manifestations, such as Kawasaki disease, this systematic review showed that MIS-C was found to be associated with an increased need for vasoactive medication. In addition, we found that MIS-C differs from Kawasaki disease in terms of inflammatory biomarkers and vascular damage biomarkers, which might explain the increased need for vasoactive medication. Previous studies showed that MIS-C is characterized by more common lymphopenia, more severe cytokine storm, and extremely high levels of N-terminal BNP, troponins, and D-dimer [41], similar to our findings. Other differences from Kawasaki disease include a presentation in older children and teenagers, less skin manifestation, less common lymphadenopathies, shock syndrome in almost all children with MIS-C, more common severe myocarditis and pericarditis, more prominent gastrointestinal symptoms, multiorgan failure, and a positive test of SARS-CoV-2 or history of positive contact [41,42].

The types and levels of inflammatory markers were suggested to help differentiate MIS-C from and exclude other diagnoses. It was found that serum inflammatory markers are increased in MIS-C compared to children with COVID-19, especially C-reactive protein, ferritin, and interleukin-6. Lymphocytopenia and thrombocytopenia were reported to be up to ten times higher in MIS-C than in COVID patients [43,44]. 

The radiographic findings in MIS-C align with affected organ systems, with features of cardiomegaly and pleural effusion, heart failure, and ascites being the most common radiographic findings [45-47]. Abdominal findings mostly include hepato-splenomegaly, gallbladder edema, mesenteric lymph nodes, intestinal wall thickening, and free fluid [46]. Similarly, this systematic review showed that cardiovascular abnormalities, visceral organs, gallbladder and bowel abnormalities, and ascites were the most common radiographic findings. This widespread abnormality on radiographic images is attributed to inflammatory spreading damaging multiple organs, which explains hyperinflammatory shock [2]. This might also explain the hypotension and shock reported among MIS-C patients. Studies showed that bilateral infiltrates are more prevalent in chest X-ray findings in MIS-C patients than ground-glass appearance or patchy shadowing, which are more prevalent in COVID-19-infected children and adults’ chest X-ray images [34].

The MIS-C treatment approaches primarily focus on immunomodulatory drugs like IVIG and glucocorticoids as initial treatment options. If unresponsiveness to IVIG and glucocorticoids occurs, a more intensive immunomodulatory approach using anakinra, tocilizumab, and infliximab is considered [41,48]. This is similar to the treatment approaches reported in the studies included in our systematic review. Our findings showed that mechanical ventilation, ECMO, and ionotropic therapy decreased mortality and hospitalization, especially in children with MIS-C in shock. This is similar to previous studies that showed that ECMO correlated with survival upon hospital discharge [10,44,48]. Moreover, the combination of ECMO, inotropic support, diuresis, COVID-19 convalescent plasma, corticosteroids, IVIG, rIL1a, and remdesivir in a critically ill MIS-C child was associated with survival and a return to their pre-illness condition [48]. It was found the most prevailing therapies are IVIG (85.6%), steroids (77.7%), and antiplatelet medications (73.7%), and the utilization of these treatments has progressively risen, especially among patients not needing PICU admission (P<0.001) [49]. 

There are some limitations for consideration. The systematic review design used is prone to selection bias and statistical heterogeneity. The included studies were diverse regarding study designs, populations, and settings, and there were no consistent criteria for evaluating variables among studies, which limited the possibilities of meta-analysis and might have affected the association reported by the included studies. Considering MIS-C is still relatively new, most studies could not establish strong conclusions based on their findings, highlighting the need for continuous research on this topic.

## Conclusions

This systematic review showed that the WHO definition of MIS-C was favored. MIS-C primarily affects male children aged 6-12 years and involves a range of systems, primarily gastrointestinal, cardiovascular, hematologic, mucocutaneous, and respiratory, due to a systemic inflammatory process. The findings unveiled elevated inflammation markers and fever as characteristic traits. Notably, this review showed that MIS-C could be distinguished from severe COVID-19 by differences in cytokine responses and autoantibody profiles. Treatment primarily involved immunomodulatory therapies like IVIG, glucocorticoids, and interleukin-6 or 1RA inhibitors, with the use of ECMO and mechanical ventilation in critically ill patients. MIS-C is associated with increased morbidity and ICU admission compared to pediatric COVID-19 patients. Therefore, multisystem involvement, especially gastrointestinal, mucocutaneous, and elevated inflammatory markers, should raise the suspicion of MIS-C, and cardiac screening should be performed. Although uncommon, MIS-C can cause serious complications and even death if not identified and treated promptly. Understanding the clinical presentation and taking a multidisciplinary approach to its therapy is critical for providing the best possible outcomes in affected children. As the medical community learns more about this disease, continuous research and collaboration are critical to improving knowledge and developing better MIS-C prevention and therapy strategies.
